# Supplementary material for: Crawling and Gliding: A Computational Model for Shape-Driven Cell Migration
Source: PLoS Comput Biol. 2015 Oct 21;11(10):e1004280. doi: 10.1371/journal.pcbi.1004280 (PMC4619082; doi:10.1371/journal.pcbi.1004280)
Supplement: S1 Code — (ZIP) [file pcbi.1004280.s012.zip › release/tst/doc/html/ca_8h.html]

Tissue Simulation Toolkit: ca.h File Reference


Tissue Simulation Toolkit
 0.1.4.1


- Main Page
- Namespaces
- Classes
- Files

- File List
- File Members

Classes

ca.h File Reference

`#include "mainpage.h"`  
`#include <vector>`  
`#include <stdio.h>`  
`#include "graph.h"`  
`#include "pde.h"`  
`#include "cell.h"`

Include dependency graph for ca.h:

This graph shows which files directly or indirectly include this file:

Go to the source code of this file.

|  |  |
| --- | --- |
| Classes | |
| class | Dir |
|  | |
| class | CellularPotts |
|  | |


---

Generated on Thu Aug 14 2014 22:04:01 for Tissue Simulation Toolkit by  

 1.8.6
